# Operando NRIXS and XAFS Investigation of Segregation Phenomena in Fe‐Cu and Fe‐Ag Nanoparticle Catalysts during CO_2_ Electroreduction

**DOI:** 10.1002/anie.202010535

**Published:** 2020-10-06

**Authors:** Sebastian Kunze, Philipp Grosse, Miguel Bernal Lopez, Ilya Sinev, Ioannis Zegkinoglou, Hemma Mistry, Janis Timoshenko, Michael Y. Hu, Jiyong Zhao, Ercan E. Alp, See Wee Chee, Beatriz Roldan Cuenya

**Affiliations:** ^1^ Department of Physics Ruhr-University Bochum 44780 Bochum Germany; ^2^ Department of Interface Science Fritz-Haber Institute of the Max Planck Society 14195 Berlin Germany; ^3^ Advanced Photon Source Argonne National Laboratory Chicago USA

**Keywords:** CO_2_ electroreduction, nanoparticles, NRIXS, operando measurements, XAFS

## Abstract

Operando nuclear resonant inelastic X‐ray scattering (NRIXS) and X‐ray absorption fine‐structure spectroscopy (XAFS) measurements were used to gain insight into the structure and surface composition of FeCu and FeAg nanoparticles (NPs) during the electrochemical CO_2_ reduction (CO_2_RR) and to extract correlations with their catalytic activity and selectivity. The formation of a core–shell structure during CO_2_RR for FeAg NPs was inferred from the analysis of the operando NRIXS data (phonon density of states, PDOS) and XAFS measurements. Electrochemical analysis of the FeAg NPs revealed a faradaic selectivity of 36 % for CO in 0.1 M KHCO_3_ at −1.1 V vs. RHE, similar to that of pure Ag NPs. In contrast, a predominant selectivity towards H_2_ evolution is obtained in the case of the FeCu NPs, analogous to the results obtained for pure Fe NPs, although small Cu NPs have also been shown to favor H_2_ production.

## Graphical abstract

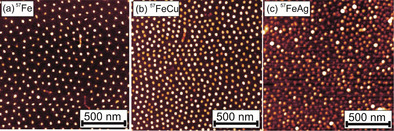

## Introduction

One of the most significant challenges in the field of catalysis is the development and optimization of experimental methods that allow the observation of a catalyst while at work.[^1^, ^2^, ^3^] This is especially difficult when nanomaterials are considered under realistic conditions, as for example, when trying to understand atomic segregation phenomena and other structural/chemical modifications at liquid/solid interfaces during an electrochemical process.^[4]^ A number of established methods are not directly applicable under reaction conditions due to decreased electronic mean free paths, scattering or lack of sufficient spatial resolution or chemical sensitivity. The development of additional techniques suitable for *operando* implementation in an electrochemical environment is therefore a subject of high interest.[^5^, ^6^]

Nuclear resonant inelastic X‐ray scattering (NRIXS) is a synchrotron radiation technique that is sensitive to vibrational states of the nuclei of Mössbauer‐active isotopes, most commonly ^57^Fe.^[7]^ It can be used to probe vibrational modes of molecular complexes of iron as well as phonons in solid state materials.^[8]^ Because the vibrational modes of a crystal lattice depend heavily on its structure, it is possible to relate NRIXS data to structural and thermodynamic properties of a material.^[9]^ Moreover, due to the isotope‐specific detection, one can probe only a specific region of a sample if enriched with the Mössbauer‐sensitive element, while the overall structure can be inferred indirectly from the contribution of the partial ^57^Fe‐phonon density of states (PDOS).[^10^, ^11^] More importantly, this method can be used to investigate a wide range of materials from bulk materials to thin‐films and NPs in experiments under extreme conditions such as high‐pressure environments or during thermal or electrochemical catalytic reactions.[^12^, ^13^, ^14^, ^15^]

In this study, the NRIXS method was used to follow the structural and chemical evolution of small ^57^Fe‐containing bimetallic NPs in a liquid environment under an applied external potential during the electrochemical reduction of CO_2_ (CO_2_RR). X‐ray absorption fine‐structure spectroscopy (XAFS), another synchrotron technique with high sensitivity to elemental and local structural composition was also applied here under reaction conditions in an electrochemical environment to complement the NRIXS insight, including providing information on the Ag and Cu components of our electrocatalysts.^[16]^

We have used iron‐based materials in our study due to their applications as heterogeneous catalysts in the field of sustainable energy conversion. Iron is an abundant metal with little environmental impact, though it is unsuitable for CO_2_RR in its bulk form, since it favors the parasitic hydrogen evolution reaction (HER).^[17]^ Nevertheless, if present in the NP form at the core of nanostructures with a CO_2_RR‐active thin shell, it could contribute to a decrease in the catalyst price. To date, research on iron‐based materials for CO_2_RR focuses mainly on molecular complexes, as for example Fe‐N‐C materials in porphyrin‐like structures.[^18^, ^19^] Here, we combine iron with copper and silver within a micellar nanoreactor since these elements are promising for the selective conversion of CO_2_ to C_1_‐C_3_ products (Cu) and CO (Ag).^[17]^ In this work, the structure and composition of the FeCu and FeAg catalysts during the reaction will be extracted from a synergistic combination of *operando* NRIXS and XAFS measurements and correlated with the selectivity trends of monometallic Cu, Ag and Fe NPs of similar size.

## Results and Discussion

**Figure 1:**
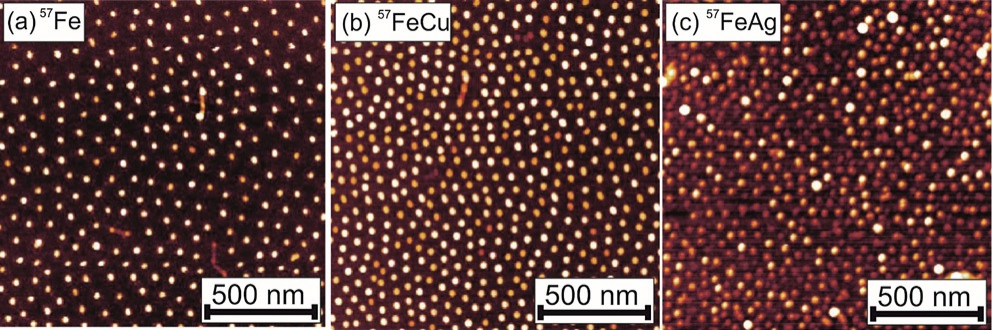
Tapping mode AFM images of: a) ^57^Fe, b) ^57^FeCu, c) ^57^FeAg NPs deposited on SiO_2_/Si(100) after a N_2_ plasma treatment for polymer removal. Average particle sizes are: 7.8 nm for ^57^Fe, 5.2 nm for ^57^FeCu and 4.2 nm for ^57^FeAg.

AFM images of the ^57^Fe, ^57^FeCu and ^57^FeAg NPs deposited on SiO_2_/Si(100) are shown in Figure 1. Additional images and particle height histograms can be found in the Supporting Information, Figures S2, S3. Well dispersed, size‐selected NPs with average size (NP height) under 8 nm were obtained with the micellar synthesis.

**Figure 2:**
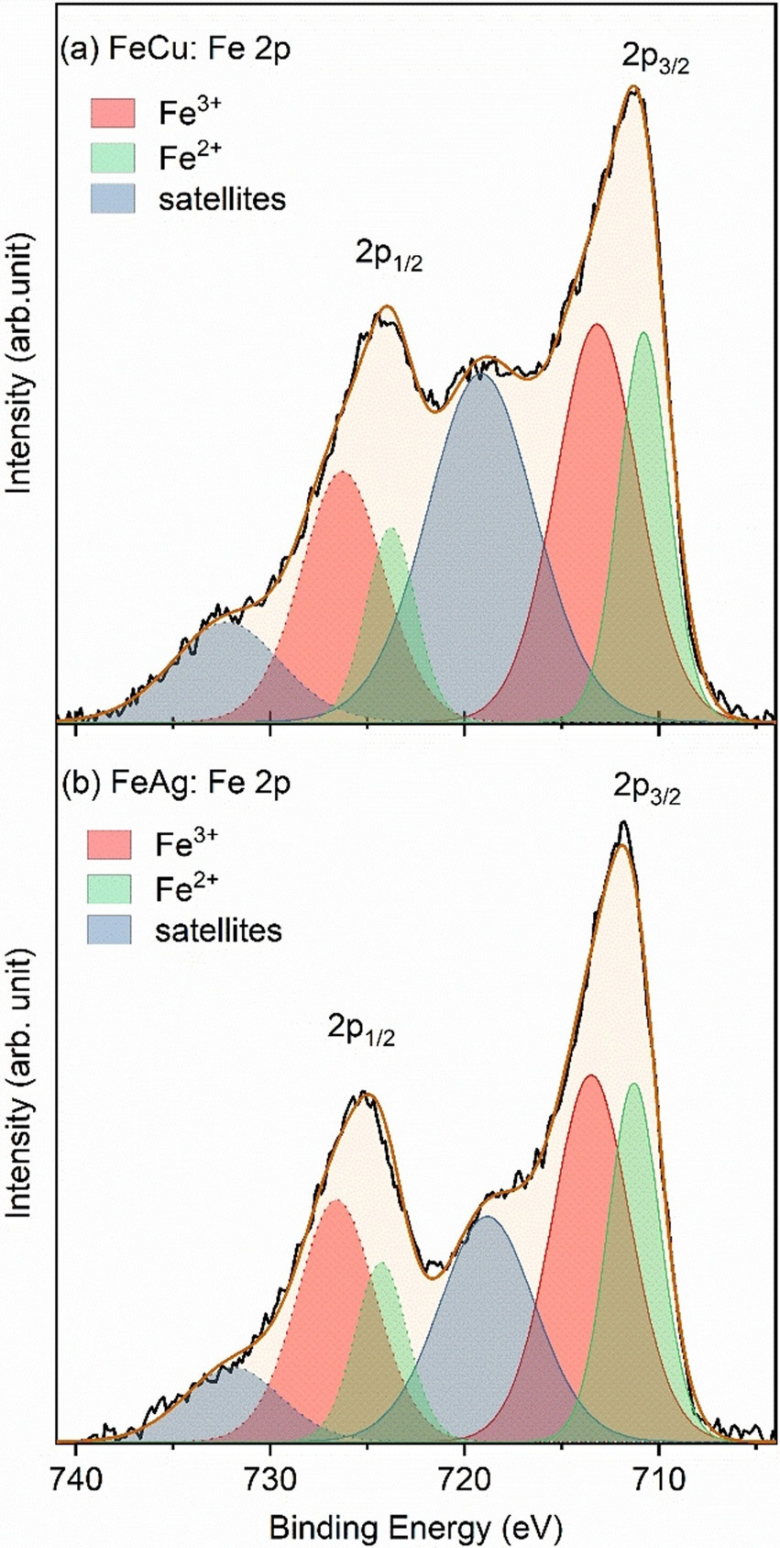
Background‐subtracted XPS spectra and corresponding fits of the Fe 2p core level region of as‐prepared a) FeCu and b) FeAg NPs supported on SiO_2_/Si(100).

XPS spectra of the Fe 2p region of the as‐prepared bimetallic NPs deposited on a SiO_2_/Si(100) substrate and the corresponding fits displaying the different Fe oxidation states are shown in Figure 2. Additional XPS data from the Cu 2p and Ag 3d regions are shown in the Supporting Information, Figure S5. It is evident that the iron in the NPs after synthesis, N_2_‐plasma treatment and subsequent air exposure is almost completely cationic (Fe^2+^ or Fe^3+^; Supporting Information, Table S1). A strong Fe 2p satellite is apparent in the FeCu spectrum, which has previously been reported for oxidized iron in the presence of copper.^[20]^ The Fe:Cu ratio was calculated to be 55:45. The Ag 3s peak in FeAg overlaps with the Fe 2p region, which we compensated for when we calculated the Fe:Ag ratio of 64:36. Owing to the small size of the nanoparticles, we expect the entire particle volume to be probed by XPS.

**Figure 3:**
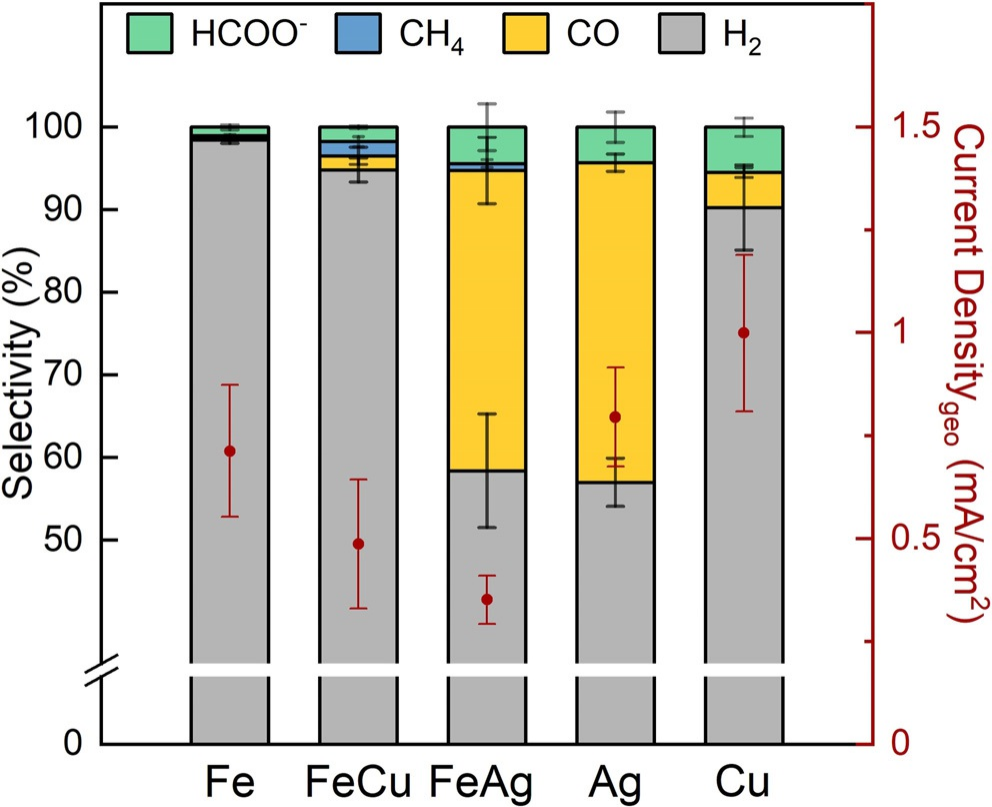
Selectivity for the reaction products of CO_2_RR obtained from Fe (6.7 nm), FeCu (5.3 nm), FeAg (4.1 nm), Ag (4.1 nm), and Cu (5.6 nm) NPs after 1 h of reaction at −1.1 V vs. RHE in CO_2_‐saturated 0.1 M KHCO_3_ and a CO_2_ flow of 20 mL min^−1^. Plotted in red are the geometric current densities of each sample (right‐hand side red scale).

The product selectivities and current densities of our Fe, FeCu, FeAg, Ag, and Cu NP samples are shown in Figure 3. While hydrogen is the main product for all samples, the selectivities for CO of the FeAg and Ag NPs are similar (36 % and 39 %, respectively) at −1.1 V vs. RHE. This similarity is consistent with a core–shell rearrangement of the FeAg NPs under reaction conditions, with Ag at the NP surface and Fe at the core. The FeCu NPs also produce CO, but with a lower selectivity of under 2 %. In contrast to FeCu, both CO and formic acid selectivities are higher in the pure Cu NP sample (4.2 % and 5.5 %, respectively). This is likely indicative of a more Fe‐rich surface in the FeCu NPs. As expected, higher hydrocarbons or alcohols were not detected in either of the FeCu or Cu samples, contrary to the case of bulk Cu. We attribute this difference to the enhanced number of low‐coordinated surface sites in small NPs that are known to strongly bind hydrogen and favor the HER.[^21^, ^22^, ^23^] Methane, in turn, is a product for all iron‐containing samples here, albeit with very low selectivity (<1.7 %), but not for pure Ag or Cu NPs. Methane production might be also affected by the NP size,^[19]^ since bulk iron is not known to yield methane.^[17]^ The methane production in the case of the FeAg samples might be explained by the presence of an incomplete (non‐uniform) Ag‐shell, leaving exposed small areas of the Fe core, which might lead to a synergistic interaction between Fe and Ag. We observed only low amounts of C_1_ products typical of bulk Cu electrodes (CH_4_, CO, HCOO^−^)^[17]^ for FeCu. The pure iron sample produced overwhelmingly hydrogen as its main product, with only traces of formic acid and methane. Besides CO and hydrogen, the silver NPs were also found to produce formic acid with 4.3 % selectivity, which is thus similar to that of the FeAg NPs (4.4 %). The current densities normalized to the geometric area of the sample for the Fe, FeCu, FeAg, Ag and Cu NP samples are: 0.71 mA cm^−2^, 0.49 mA cm^−2^, 0.35 mA cm^−2^, 0.79 mA cm^−2^ and 0.99 mA cm^−2^ respectively. A possible explanation for the lower current density, as well as slightly lower CO selectivity of the FeAg sample as compared to pure Ag, is the compressive strain induced by the smaller iron core on the silver overlayer. A shift of the d‐band center away from the Fermi level, caused by compressive strain, is expected to influence the CO bonding strength negatively, leading to a more favorable H_2_ production.

**Figure 4:**
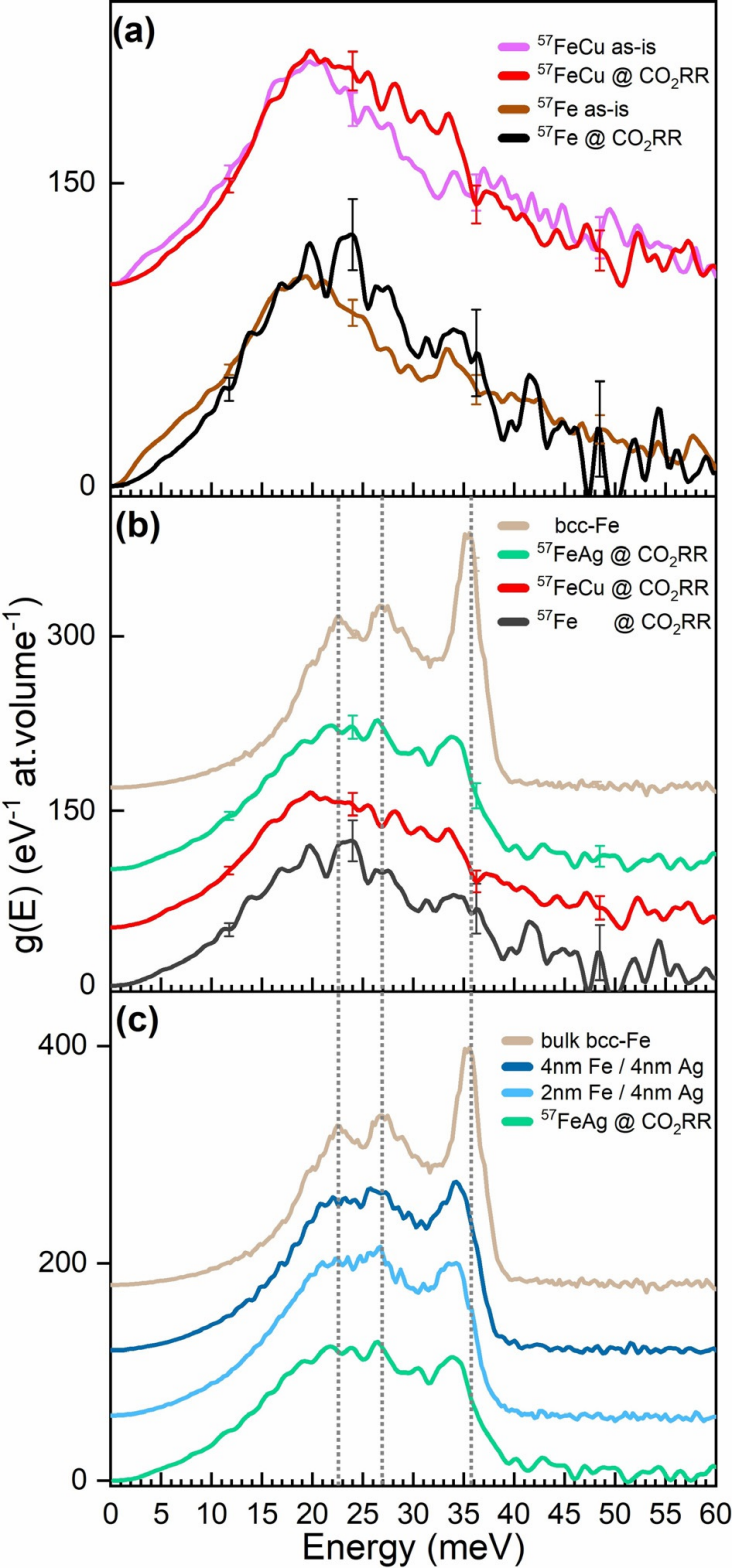
a) ^57^Fe‐partial PDOS, g(E), obtained from raw NRIXS spectra of ^57^Fe and ^57^FeCu NPs in air and under CO_2_RR. b) The *operando* CO_2_RR PDOS data of ^57^Fe, ^57^FeCu, and ^57^FeAg NPs together with those for a bulk bcc‐Fe reference foil for comparison.^[12]^ c) ^57^Fe‐partial PDOS of ^57^FeAg NPs plotted together with thin ^57^Fe layers deposited on a Ag film (4 nm) reproduced from Ref. [12]. Representative error bars in different regions of the spectra are also shown in (a) and (b). The spectra have been vertically offset for better visibility and the vertical lines indicate the position of the two TA and the LA peaks of bulk bcc‐Fe.

NRIXS measurements were carried out before (^57^Fe, ^57^FeCu in air) and *operando* under CO_2_RR conditions in the electrolyte and under potential control (^57^Fe, ^57^FeCu, and ^57^FeAg). Figure 4 shows the corresponding Fe‐partial PDOS of these samples. The spectrum from a bulk bcc‐Fe foil is also shown for reference. The spectra recorded in air indicate enhanced atomic disorder and oxidic Fe features at 41 meV −43 meV, that are at least partially reduced under reaction conditions, Figure 4 a. The longitudinal acoustic (LA) phonon peak near 36 meV observed in bulk bcc‐Fe becomes also visible in the NP phonon spectra under reaction conditions. Nevertheless, it appears shifted to lower phonon energies (by 1.3 meV to 2 meV; Supporting Information, Table S2) in both of the bimetallic samples due to the presence of the phononically softer Ag and Cu metals in the Fe environment. Furthermore, significant damping is observed in the LA peak of the three NP samples as compared to that of bulk bcc‐Fe due to size‐effects.

Interestingly, the PDOS of the ^57^FeAg NP sample shows the most clear resemblance to that of bulk bcc‐Fe, since for that sample the LA peak is more prominent and we also see the transverse acoustic (TA) phonon modes, enhanced ordering (as indicated by sharper features), and the absence of oxidic species under the reducing conditions available during CO_2_RR. Such observations point towards a significant reduction of the FeO_x_ species when the potential is applied in parallel to the segregation of Ag to the NP surface and the formation of a protective Ag shell and a pure bcc‐Fe NP core.

For the pure ^57^Fe NPs we still observe hints of the incomplete FeO_*x*_ reduction under reaction conditions and enhanced disorder (smeared lines). The ^57^FeCu NPs look more disordered and the Fe‐partial phonon DOS has a larger deviation with respect to that of pure bcc‐Fe (Figure 4 b), indicating some degree of Fe‐Cu intermixing. Nevertheless, a Cu‐rich surface is still expected since the PDOS of this sample is still in close agreement with that of pure bcc‐Fe aside from the size‐dependent phonon damping and enhanced disorder. Such spectra are also seen for thin Fe layers in the proximity of metals with a lower phonon cut‐off energy, as is the case here for both, Cu and Ag.^[14]^ Overall, the PDOS corresponding to the FeAg and FeCu NP samples acquired under CO_2_RR approaches that of a bcc‐Fe structure, not fcc‐Fe as could be expected if Fe would be embedded in the fcc‐Cu or fcc‐Ag matrixes.^[24]^ It should be noted that according to XPS the relative content of Fe in the as prepared FeCu and FeAg NPs is similar, namely 55 % and 64 %.

The Fe/Ag is a layered system known for a low interfacial intermixing. The PDOS of the NPs in this sample displays analogous characteristics to that of a Fe/Ag multilayer interface which was investigated by Roldan et al.^[12]^ Figure 4 c shows the Fe‐partial PDOS data of this FeAg NP sample alongside the previously reported data from Fe/Ag multilayers.^[12]^ Apart from the overall shape and correspondence to a bcc structure, the positions of the LA peak around 35.5 meV and the TA peaks (20–30 meV) of the FeAg NPs are similar to those of the sandwiched Fe/Ag layers with Fe thicknesses of 2–4 nm. This is in accordance with the average particle size of the FeAg sample of about 4.2 nm (the real size of the iron part is smaller than this owing to the bimetallic composition).

A comparison of the PDOS of the ^57^FeCu NP sample under working conditions and that of Fe(1.5 nm)/Cu(4 nm) multilayers from Roldan et al.^[12]^ is displayed in Figure S6. While the LA peaks of the multilayer sample and those of the ^57^FeCu NPs under working conditions are similarly positioned (34.1 meV vs. 33.7 meV) and shaped, corresponding to the bcc‐Fe structure, there is a lack of unambiguous structural features in the TA region, indicating high disorder due to the small NP size and phonon softening caused by the Fe‐Cu interaction.

XAFS data were also acquired under CO_2_RR conditions using the same cell design and samples as in the NRIXS experiments in order to extract complementary information about the chemical state and structure of the samples, including their changes under reaction conditions. In this case, we could gain access not only to the Fe‐component of the bimetallic systems, but also to the Ag and Cu constituents.

**Figure 5:**
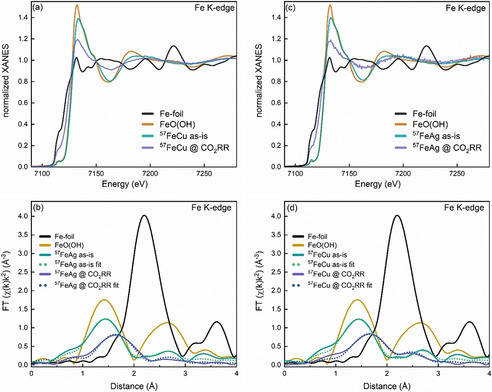
Fe K‐edge XANES (a,c) and Fourier‐transforms of k^2^‐weighted EXAFS data (phase‐uncorrected) and the corresponding first‐shell fits (dotted lines) (b,d) of ^57^FeCu and ^57^FeAg NPs as prepared (as‐is) and under CO_2_RR conditions at −1.1 V vs. RHE in 0.1 M KHCO_3_ after 3.5 h (^57^FeCu) and 1.5 h (^57^FeAg). Reference spectra from lepidocrocite FeO(OH)^[37]^ and a bulk iron foil are also shown for comparison.

It is evident from the XANES spectra in Figure 5 a,c that the particles are in an oxidized state from the beginning, as they show typical oxidic features. For comparison with our oxidized particles, we chose here to show the spectrum for an iron mineral (lepidocrocite) that consists of a naturally occurring hydroxide/oxide mixture, since the initial particle composition after air exposure is likely a complex mixture as well. From Figure 5 b,d we can conclude that the first coordination shell of our oxidized NP samples is similar to that in the reference mineral, but the mean Fe−O distance of 1.81±0.06 Å in our samples is smaller than the reference value of 2.00 Å.^[25]^ Table 1 summarizes further relevant parameters extracted from the EXAFS measurements, both for as‐prepared samples and samples under CO_2_RR conditions.

**Table 1:** Coordination numbers (CN) and interatomic distances (R), derived from the fit of EXAFS data of samples ^57^FeCu and ^57^FeAg at the Fe, Cu, and Ag K‐edges.^[a]^

Sample	Treatment	CN	R [Å]	CN	R [Å]	CN	R [Å]	CN	R [Å]
		Cu‐O		Cu‐Cu		Fe‐O		Fe‐Fe	
^57^FeCu	As is	2.3(3)	1.95(1)			6.9(2.1)	1.81(6)						
	Operando	0.7(6)	1.95(3)	4.2(1.2)	2.56(1)	2.3(1.2)	1.81(4)	1.5(0.9)	2.48(5)				
		Ag‐Cl		Ag‐Ag		Fe‐O		Fe‐Fe	
^57^FeAg	As is	4.6(6)	2.81(1)			6.7(1.6)	1.81(2)						
	Operando			5.4(7)	2.941(1)	2 (1)	1.81(4)	1(1)	2.48(6)				
									
Bulk	Cu foil^[33]^			12	2.566								
	CuO^[34]^	4	1.95(1)										
	Ag foil^[35]^			12	2.883(7)								
	AgCl^[36]^	6	2.79(1)										
	FeO(OH)^[25]^					6	2.00						
	Fe foil^[26]^							8+6	2.47(1)

[a] The data were measured in air (as‐is) and *operando* during CO_2_RR at −1.1 V vs. RHE in 0.1 M KHCO_3_ after 1.5–3.5 h. The uncertainty of the last digit is given in parentheses. Additional parameters and fit results are shown in the Supporting Information, Tables S3 and S4. A single Fe−Fe scattering path was used to fit the overlapping contributions from the first two coordination shells in bcc Fe owing to the limited resolution in R‐space as a result of the short Fe K‐edge spectrum.

Upon applying a potential of −1.1 V vs. RHE, the particles start reducing to a more metallic state. However, we still see a significant contribution from oxidized iron species under reaction conditions. Some of these cationic species might also be assigned to iron compounds dissolved into the electrolyte.

We can show a reduction and increase in crystalline order in the iron parts of the NP samples under reaction conditions that are in agreement with our previous NRIXS results. The operando XAFS measurements were acquired over periods of 1.5–3.5 h of the reaction, while the NRIXS data were acquired over periods of 11 h–18 h, which might also explain the higher apparent content of iron oxides in the XAFS data. The long acquisition times were needed due to the low count rates in the NRIXS experiment.

**Figure 6:**
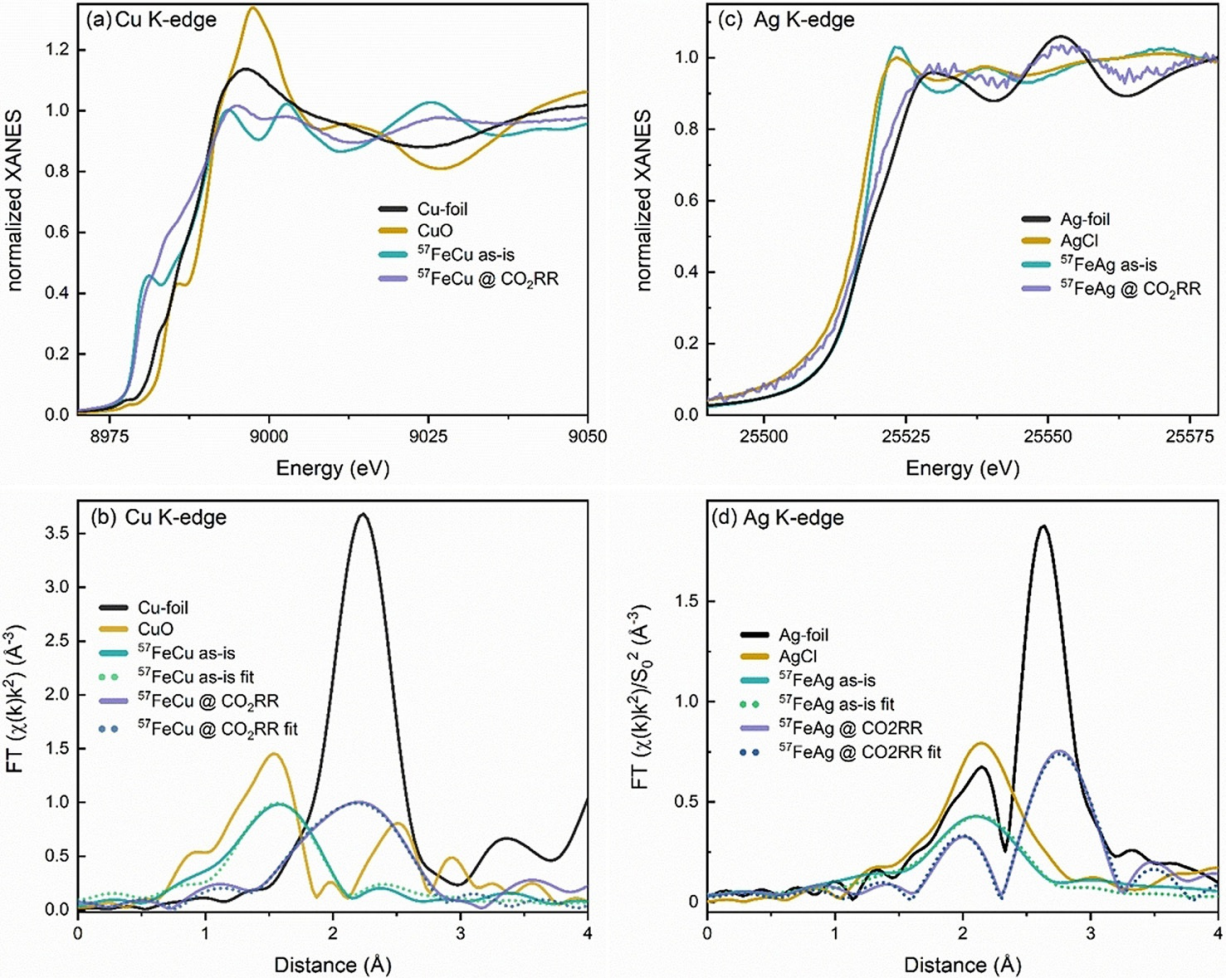
Cu K‐edge (a,b) and Ag K‐edge (c,d) XANES (a,c) and Fourier‐transforms of k^2^‐weighted EXAFS data (phase‐uncorrected) (b,d) of ^57^FeCu and ^57^FeAg NP samples in as‐prepared state (as‐is) and under CO_2_RR conditions (−1.1 V vs. RHE in 0.1 M KHCO_3_ after 1.5 h (^57^FeCu) and 2.5 h (^57^FeAg)) Reference spectra from CuO, Cu‐foil, AgCl^[37]^ and a Ag‐foil are shown for comparison.

Figure 6 displays Cu K‐edge (a,b) and Ag K‐edge (c,d) XANES and EXAFS data of the ^57^FeCu and ^57^FeAg NP samples. The presence of oxidic copper compounds similar to CuO was observed, and a reduction of copper to the metallic state under *operando* conditions. The average interatomic distance for Cu−O in the as‐prepared sample is in line with that of bulk CuO (Table 1). The time dependence of the reduction process (Supporting Information, Figure S7) reveals a fast initial CuO reduction, followed by a slower increase over the next 1.5 h of the peak in the Fourier‐transformed (FT) EXAFS at ca. 2.2 Å that corresponds to a contribution of the metallic Cu phase. For the as‐synthesized samples, both XANES and EXAFS data collected at the Ag K‐edge and shown in Figure 6 c,d, indicate the presence of stable AgCl compounds due to residual Cl from the synthesis procedure. Upon applying the CO_2_RR potential, a clear increase of the metallic silver contribution (peak in in the FT‐EXAFS at ca. 2.7 Å) is seen, and no other components could be fitted. There is no contribution from Fe−Ag scattering paths, and the Fe−Fe and Ag−Ag distances do not hint towards intermixing of Fe and Ag either, since they are in line with those for the corresponding bulk materials. In the case of the ^57^FeCu sample, the Cu−Cu distance agrees with that in bulk Cu, while the Fe−Fe distance of 2.48±0.05 Å is in line with that of bulk Fe at 2.47±0.01 Å.^[26]^

After careful evaluation of the *operando* structural and chemical information extracted from two complementary synchrotron X‐ray methods (XAFS and NRIXS), we demonstrate a significant reduction of iron oxide to metallic species under reaction conditions, accompanied by a structural transformation from an amorphous or atomically disordered phase to a crystalline structure. We observed a similar transformation for the secondary metals in the bimetallic NP samples via XAFS; a clear reduction of CuO to Cu in ^57^FeCu, and even more pronounced, AgCl to Ag transformation in ^57^FeAg. NRIXS served to distinguish segregated Fe‐Ag and Fe‐Cu phases (the present case) from alloyed structures, since we only detected a bcc‐like Fe structure typical for a segregated Ag shell with bcc‐Fe NP core. A small degree of Fe‐Cu intermixing is obtained for the ^57^FeCu sample from the XAFS data analysis. The bcc‐Fe phase also predominates the NRIXS signal. Both these findings suggest the predominant segregation of both metals in our samples under CO_2_RR conditions.

It should be noted that the X‐ray‐based spectroscopic methods employed here for the characterization of our nanosized electrocatalysts (NRIXS and XAS) also have limitations that need to be considered for the interpretation of the data obtained. For instance, both methods are bulk‐sensitive ensemble‐averaging techniques, that is, the measured signal is an average of contributions from all metal species, residing both at the surface and in the core regions of all NPs within the X‐ray irradiated sample area. Therefore, meaningful composition information can only be obtained when applied to multimetallic samples with a homogeneous inter‐ and intraparticle composition and chemical state. Furthermore, the interpretation of structural characteristics extracted from X‐ray spectroscopy methods will be further hindered when broad nanoparticle size and shape distributions exist in the as prepared samples.[^27^, ^28^] This is even more relevant when *operando* catalysis studies are undertaken, since in that case the coexistence of particles of different size and shape evolving under reaction conditions is common. Therefore, the interpretation of structural information extracted from XAS and NRIXS in terms of 3D structural motifs for inhomogeneous systems should be carried out with extreme care and must rely on complementary insight from additional techniques such as microscopy methods and theoretical modelling.

In the present study, we combined AFM, TEM, XPS, NRIXS, and XAS to gain insight into the evolution of the structure and composition of homogeneously dispersed size and shape‐controlled FeCu and FeAg NPs during CO_2_RR. We remark here that for the FeAg NPs, the EDX line profiles for Ag acquired after reaction (Supporting Information, Figure S4) extend further outwards as compared to the Fe profiles, which suggests some degree of Ag segregation to the surface. For the FeCu NPs, the line profiles indicate more even mixing of the two elements. These STEM‐EDX results are, however, limited due to the difficulty of obtaining high quality and contaminant‐free images in the presence of the Nafion NP binder and carbon support, which strongly interacts with the electron beam, resulting in the need of employing short acquisition times. Nevertheless, the microscopy data are consistent with the X‐ray spectroscopy and selectivity data regarding the segregation behavior in the FeCu and FeAg NPs.

According to the respective surface energies[^29^, ^30^, ^31^] and atomic sizes, the formation of a Cu or Ag shell and a bcc‐Fe core is expected. For instance, we have previously shown the segregation of copper to the surface of CuNi NPs under reducing conditions (CO_2_+CO+H_2_).^[32]^ This is in good agreement with the electrochemical behavior observed here for the FeAg NPs, which was found to be reminiscent to that of pure silver. The trend was not as clear for the FeCu sample, where size effects already demonstrated for pure Cu NPs will also lead to an increase of the H_2_ production with decreasing NP size at the expense of CO and hydrocarbons. Therefore, for the Fe‐Cu system, we cannot discard the possibility of having Cu‐rich and Fe‐rich regions coexisting at the NP surface, which would also explain the increased H_2_ production (taking place over the exposed Fe component) as compared to the selectivity obtained in similarly sized monometallic Cu NPs.

Although the present nanosized catalysts do not display an outstanding activity or selectivity for CO_2_RR, in line with previous studies for small NPs which also favor H_2_ evolution,[^21^, ^22^, ^23^] our study serves to provide fundamental insight into the dynamic behavior of electrocatalysts under reaction conditions. Furthermore, we illustrate a powerful combination of X‐ray based synchrotron techniques for the characterization of the structural and chemical evolution of electrocatalysts that, when available hand in hand with reactivity data, allows one to gain in depth understanding of the structural motifs and chemical compositions responsible for specific selectivity trends.

## Conclusion

This study demonstrates the dynamic transformations undergone by FeCu and FeAg NPs during the electrochemical reduction of CO_2_. In particular, *operando* NRIXS and XAFS data revealed the formation of an Ag shell surrounding a bcc‐Fe core. On the other hand, more intermixing was observed for the FeCu NPs, and the presence of separated Fe and Cu regions on the NP surface under reaction conditions could not be excluded. Interestingly, a similar CO production was observed for the thin Ag‐shell in the FeAg NPs as compared to pure Ag NPs, which indicates the optimization of the use of the noble metal. Overall, and due to the small NP size, the production of H_2_ was, however, favored, especially in the Fe and FeCu samples.

Finally, our work emphasizes that *operando* experiments are a very valuable tool to link catalytic properties to structure and composition of electrocatalysts under realistic working conditions.

## Conflict of interest

The authors declare no conflict of interest.

## Supporting information

As a service to our authors and readers, this journal provides supporting information supplied by the authors. Such materials are peer reviewed and may be re‐organized for online delivery, but are not copy‐edited or typeset. Technical support issues arising from supporting information (other than missing files) should be addressed to the authors.

Supplementary
