# Supplementary material for: Operando NRIXS and XAFS Investigation of Segregation Phenomena in Fe‐Cu and Fe‐Ag Nanoparticle Catalysts during CO2 Electroreduction
Source: Angew Chem Int Ed Engl. 2020 Oct 6;59(50):22667–74. doi: 10.1002/anie.202010535 (PMC7756314; doi:10.1002/anie.202010535)
Supplement: Supplementary file 1 — Supplementary [file ANIE-59-22667-s001.pdf]

Angewandte Chemie
Eine Zeitschrift der Gesellschaft Deutscher Chemiker

Supporting Information

Operando NRIXS and XAFS Investigation of Segregation Phenomena in Fe-Cu and Fe-Ag Nanoparticle Catalysts during CO_2 Electroreduction

*Sebastian Kunze, Philipp Grosse, Miguel Bernal Lopez, Ilya Sinev, Ioannis Zegkinoglou, Hemma Mistry, Janis Timoshenko, Michael Y. Hu, Jiyong Zhao, Ercan E. Alp, See Wee Chee, and Beatriz Roldan Cuenya**

anie_202010535_sm_miscellaneous_information.pdf

Supporting Information

Synthesis and Preparation

Inverse micelle encapsulation was used to prepare colloidal solutions of size-selected, isolated Fe, FeCu, FeAg, Ag and Cu NPs.[1-2] Two sets of NPs were synthesized by loading a nonpolar/polar diblock copolymer [poly(styrene)-block-poly-(2-vinylpyridine), Polymer Source Inc.] dissolved in toluene with metal salts ($^{57}FeCl_2$ x 2 H_2O, $AgNO_3$, $CuCl_2$ x $2H_2O$, $FeCl_3$). One of the NP sets synthesized contained the isotopically enriched $^{57}FeCl_2$-salt needed for the NRIXS measurements. The enriched iron salt was synthesized from an iron foil with 95 % ^{57}Fe isotopic enrichment by adapting a procedure described in the literature.[3] A 0.2 metal loading (metal/P2VP molecular weight ratio) was used in the synthesis of the micellar samples. Samples for NRIXS and XAFS measurements (^{57}Fe, $^{57}FeCu$, $^{57}FeAg$) were prepared by impregnating the NP solution on carbon black powder (5 wt%). A N_2-plasma treatment was used for polymer removal on the impregnated powders (300 mTorr, 20 W, 5 cycles of 10 min duration) as well as on samples dip-coated on silicon wafers used for the AFM analysis (300 mTorr, 20 W, 20 min). The NP powder (5 mg – 10 mg) was then dispersed in an ethanol/nafion solution. The former solution was deposited on a low-porosity carbon paper disc (Sigracet® SGL 24 AA, SGL Carbon GmbH) by filtration, during which the catalyst powder stays on one side, bonded by nafion, while the other side is not modified. These carbon paper samples were used in order to minimize the signal attenuation for *operando* NRIXS and XAFS measurements, since our cell is designed such that the sample is irradiated from the back, and the subsequent fluorescence signal is also collected from the back. Finally, Fe, FeCu, FeAg, Ag and Cu samples were prepared for electrochemical characterization in our laboratory by two successive cycles of dip-coating the bimetallic NP solution (nafion-free) on fresh glassy carbon electrodes and subsequent a N_2 plasma treatment (300 mTorr, 20 W, 2x20 min) was employed for polymer removal.

The NP synthesis procedure is analogue for isotopically enriched and non-enriched salts. For the synthesis of the ^{57}Fe NPs the following procedure was used. 50 mg of PS-P2VP (P4925-S2VP from Polymer Source Inc., M_{PS}=102000, M_{P2VP}=97000) were mixed with 5 mL toluene (Fischer Scientific, p.a.) and stirred overnight, leading to the formation of reverse micelles. In parallel,

7.56 mg of a $^{57}FeCl_2 \times 2\ H_2O$ salt (salt-to-polymer loading of 0.2) were mixed with 5 mL toluene and vigorously stirred overnight. Subsequently, the metal salt solution was added to the micellar polymeric solution and stirred for 72 h.

The ^{57}FeCu NPs were analogously synthesized, employing 3.78 mg of the $^{57}FeCl_2 \times 2\ H_2O$ salt and 3.96 mg of $CuCl_2 \times 2\ H_2O$. For the synthesis of the ^{57}FeAg NPs, 3.78 mg $^{57}FeCl_2 \times 2\ H_2O$ and 3.94 mg of $AgNO_3$ were used.

In order to produce the $^{57}FeCl_2 \times 2\ H_2O$ salt described above we have adapted a procedure described in the literature.[3] Concentrated hydrochloric acid and sodium bicarbonate (both p.a.) were purchased from Sigma-Aldrich. 260 mg of a ^{57}Fe foil were put into a Schlenk tube under inert gas flow and 5 mL HCl (37 %) was added in steps of 1 mL. The reaction mixture was vigorously stirred and kept at 72 °C for 7 h. Subsequently, the PTFE tube and cap were removed and replaced by a distillation bridge with an attached 50 mL flask. The distillation bridge was also connected to a vacuum line. Rapid evaporation of HCl upon opening the vacuum line was observed. After the HCl was evaporated, the temperature was slowly increased to 80 °C until all liquid was evaporated. A 350 mg residue formed on the bottom of the flask, gradually changing color from green to a very pale blue. In terms of $^{57}FeCl_2 \times 2\ H_2O$, this corresponds to 63 % of the theoretical yield, which is well within the range reported in the literature (70 %).[3]

Sample Characterization

Tapping Mode atomic force microscopy (AFM) (Bruker, Multimode 8) images were acquired on samples supported on SiO_2/Si(100) after polymer removal. The apparent NP height was used to determine the NP size and distribution. We used the Gwyddion software package to analyze the height maps.[4] STEM-EDX measurements of supported micellar NPs, dispersed in ethanol and drop-casted on 5nm amorphous Si TEM windows, were carried out in a JEOL ARM 200F microscope. We employed our XPS setup (SPECS GmbH, a non-monochromatic source, Al K-edge @ 1486.6 eV) to determine the different metal ratios in the bimetallic NPs.

Electrochemistry

The laboratory-based CO_2RR characterization was carried out in a gas-tight H-type cell. A Selemion ion exchange membrane (AGC Engineering Co., Ltd.) separated the two compartments, which were filled with 40 mL 0.1 M $KHCO_3$ (Sigma Aldrich, 99.7 %). A three-electrode setup with a platinum mesh counter electrode (MaTeck, 3600 cm^{-2}), leak-free Ag/AgCl/3.4 M KCl reference (Innovative Instruments Inc.) and the glassy carbon sample acting as the working electrode (4.4 cm^2 exposed area) were employed. A CO_2 flow of 20 mL/min was used during the measurements and for purging. The potential was controlled by a Metrohm-Autolab (M204) potentiostat with corrected iR drop. Gas analysis was done online by a Shimadzu 2014 Gas-Chromatograph with HayesSepQ + HayeSepR packed columns and a flame ionization detector (FID) for hydrocarbon separation and detection. An MS-13X column and thermal conductivity detector (TCD) were used for the detection of H_2, N_2 and O_2, while CO and CO_2 were methanized before detection by the FID. Liquid products such as formate were analyzed with a high-performance liquid chromatograph (Shimadzu Prominence HPLC) with a NUCLEOGEL SUGAR 810 column and a refractive index detector (RID). Alcohols were analyzed with a liquid GC (Shimadzu 2010 plus) with silica column and FID. Product selectivities were calculated with product distribution and current data obtained after 1h of reaction.

Synchrotron Measurements

Operando XAFS and NRIXS measurements were performed in a home-made electrochemical cell (**Fig. S1**). During both measurements, CO_2 was dosed to the electrolyte (0.1 M $KHCO_3$) solution (20 mL/min) and a potential of -1.1 V vs RHE was applied. The same three-electrode setup as described above was employed. A potentiostat EmStat 3 from PalmSense was used to control the applied potential. The XAFS experiments were performed at the SAMBA beamline at the SOLEIL synchrotron. Data at the K-edges of Cu, Fe and Ag were collected, using a Si(220) monochromator for energy selection. The measurements were performed in fluorescence mode using a 13-channel Ge detector. The NRIXS experiments were performed at the 3-ID-B beamline at the Advanced Photon Source (APS) of the Argonne National Laboratory. The beamline features a monochromatic beam at 14.41 keV with an energy resolution of 1 meV. Analysis of the NRIXS data was done with the PHOENIX software from W. Sturhahn.[5] XAFS data were processed and

analyzed with the ATHENA and ARTEMIS modules of the Demeter software package, using a FEFF6 code.[6]

Operando XAFS and NRIXS setups

The electrochemical cell is made of polyacrylic glass and enables measurements with continuous gas flow. A schematic representation is shown in **Fig. S1**. The cell has fittings for a gas inlet and gas outlet as well as for a reference electrode (RE) and a counter electrode (CE) in the electrolyte compartment. The reference electrode was a leak-free Ag/AgCl electrode (3.4 M KCl), the counter electrode a platinum mesh. The sample was mounted on the flange opening at the side by attaching its backside to kapton tape. Silicone glue was used to seal the tape and make it leak tight. A platinum wire was used to make an electric connection to the sample (the working electrode (WE)). All flanges were fixed into position with PTFE screws.

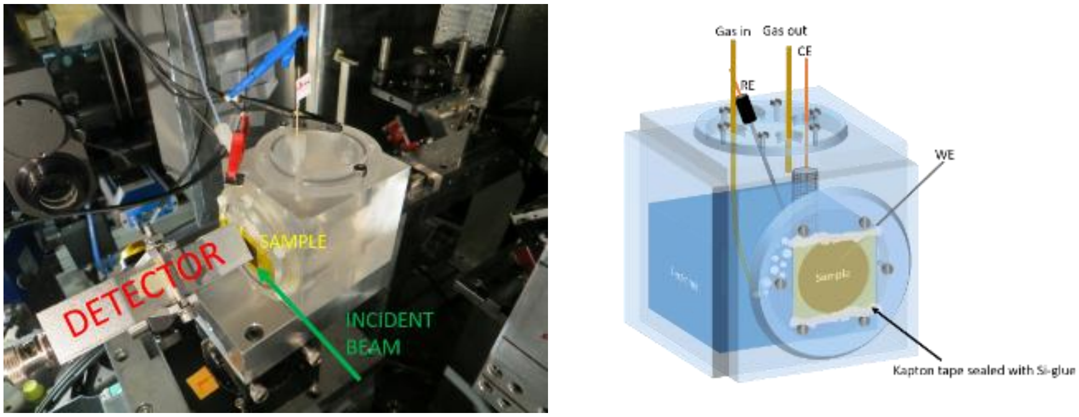

Figure S1 Experimental setup for operando measurements. Left: Detector setup for NRIXS measurements. Right: Schematic view of the polyacrylic reaction cell.

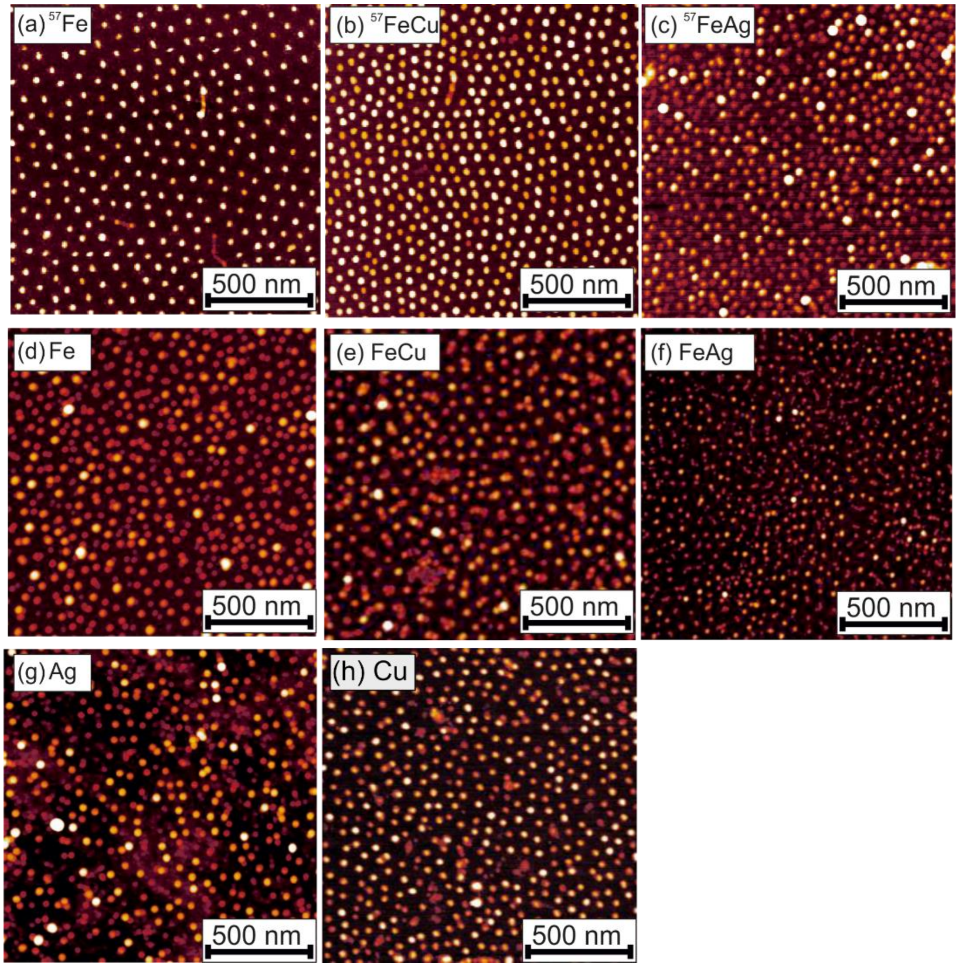

Figure S2 Additional *ex situ* AFM images of the mono and bimetallic NPs**.**

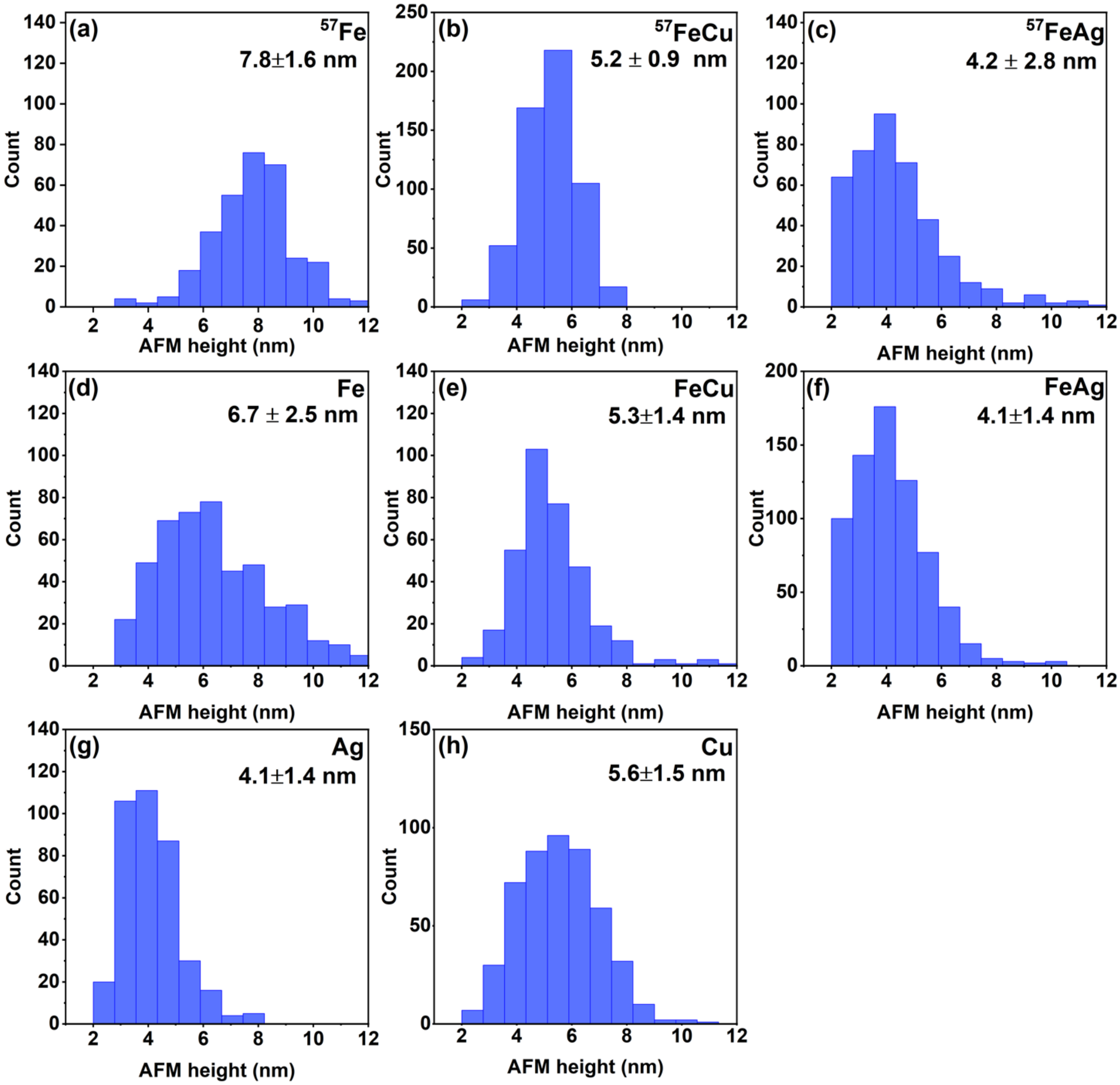

Figure S3 Nanoparticle height distributions extracted from AFM data**.** The average sizes and standard deviations are given in inserts.

STEM-EDX

STEM-EDX measurements of supported micellar NPs, dispersed in ethanol and drop-casted on 5nm amorphous Si TEM windows (from the supplier TEMwindows), were carried out in a 200 kV JEOL ARM 200F microscope. Pristine "as-prepared" NPs (deposited and calcinated on nanocrystalline SiO_2) and NPs that were used in the CO_2RR synchrotron experiments (deposited on carbon black powder and bonded with Nafion) were measured. The latter samples were scratched off from the carbon paper electrode, and then drop-casted on the TEM grid. **Fig. S4** shows images and EDX line scans of the FeCu and FeAg before and after CO_2RR.

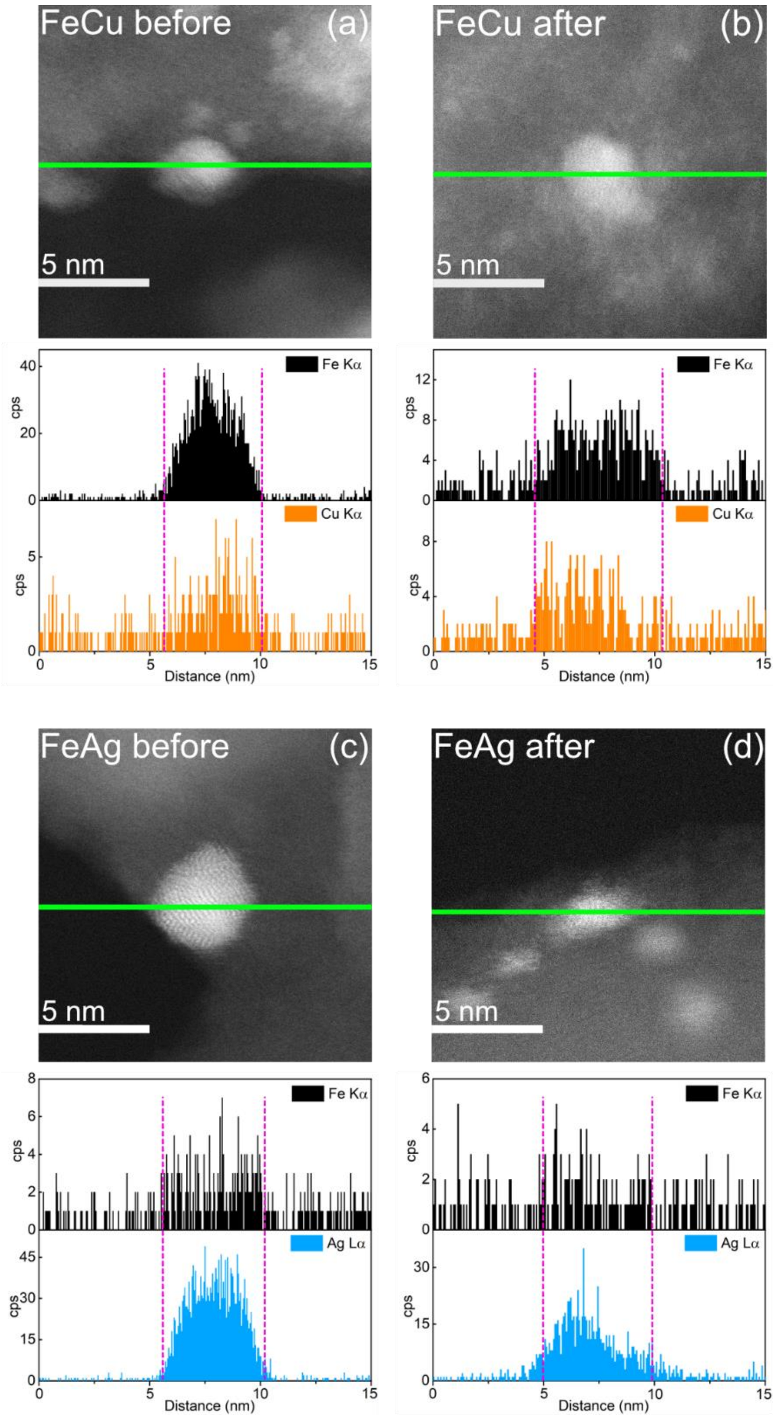

Figure S4: STEM images and EDX line cans (green horizontal lines) of micellar FeCu and FeAg NPs supported on nanocrystalline SiO_2 (a and c) and on carbon powder (b and d). Images (a) and (c) were obtained before the reaction and (b) and (d) after CO_2RR. Violet lines are reference lines to guide the eye.

Fitting of XPS spectra

All binding energies were referenced against the 2s peak of SiO_2/Si(100). For peak fitting, Shirley backgrounds were used, and Gaussian-Lorentzian (GL) line shapes.

Quantitative information of the ratio between different elements and for one element the relative contribution of different oxidation states were extracted from the analysis of the respective areas in the XPS spectra. The area of each element was previously corrected with the relative sensitivity factors RSF (tabulated values specific for each XPS instrument).

Table S1 (a) Metal ratios for pristine FeAg NPs and FeCu NPs as calculated from XPS. (b) Content of Fe^{2+} and Fe^{3+} in the as-prepared NPs.

(a)

**Sample**	**Fe**	**Cu**	**Ag**
**FeAg**	64%	-	36%
**FeCu**	55%	45%	-

(b)

**Fe ox. state**	at% in FeCu	at% in FeAg
**Fe^{3+}**	58.7%	64.1%
**Fe^{2+}**	41.3%	35.9%

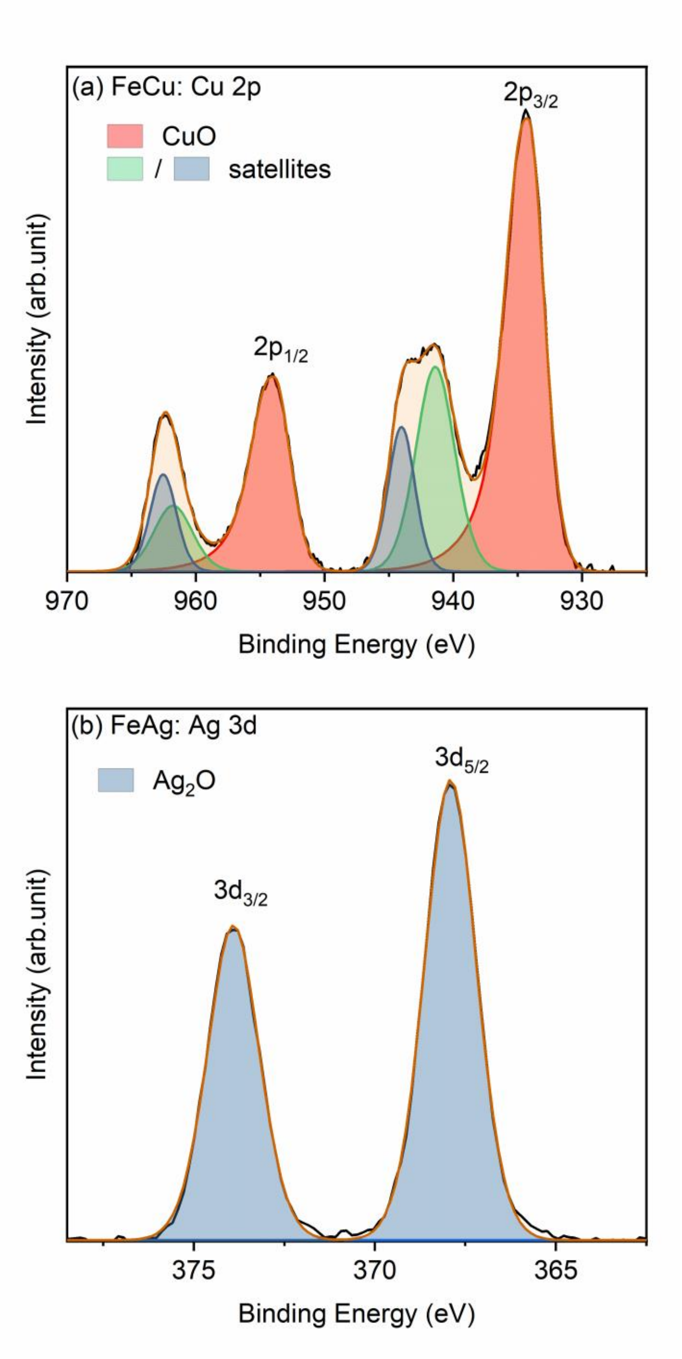

Figure S5 Background-subtracted XPS spectra of the (a) Cu-2p and (b) Ag-3d core level regions of FeCu and FeAg NPs deposited on SiO_2/Si(100).

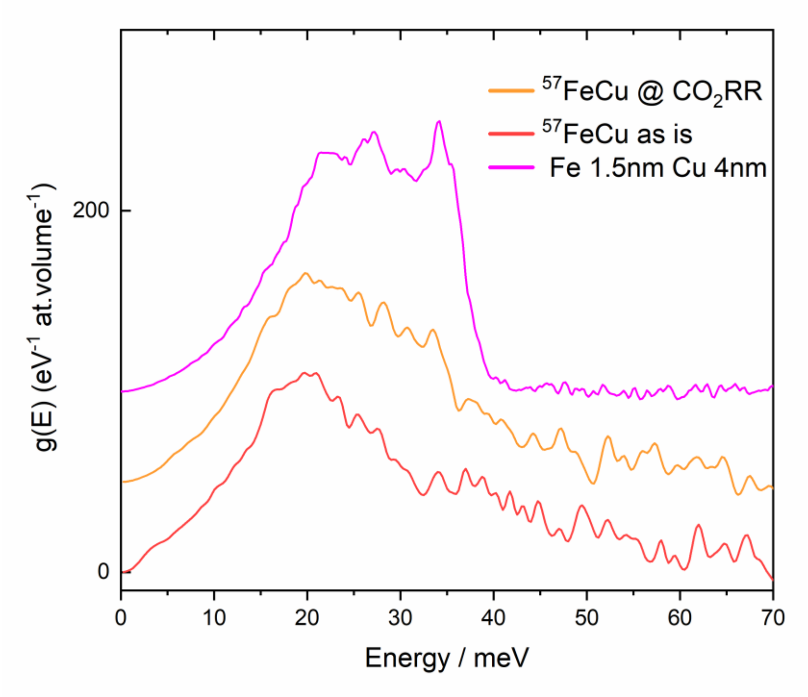

Figure S6: ^{57}Fe-partial PDOS of ^{57}FeCu plotted together with 1.5 nm thin Fe layers deposited on Cu films (4nm) reproduced from Roldan *et al*.[7] The spectra were vertically offset for better legibility.

Table S2: Summary of the position of the longitudinal acoustic (LA) peak position of ^{57}Fe, ^{57}FeCu and ^{57}FeAg and their respective shift related to bulk bcc-Fe in air and under reaction conditions.

**Sample**	**^{57}Fe**	**^{57}FeCu**	**^{57}FeAg**	**bcc-Fe**
As prepared (meV)	33.5	34.1		35.5
Operando (meV)	34.5	33.7	34.2	

EXAFS fitting

All EXAFS spectra shown are displayed phase-uncorrected. Fitting was performed using the Artemis software.[6] Photoelectron scattering phases and amplitudes were calculated using the built-in FEFF-6 code[8] and employing bulk metal models to simulate the phases and amplitudes of the metal-metal bonds in the reduced samples, CuO and FeO(OH) models to simulate the phases and amplitudes for the metal-oxygen bonds, and an AgCl model for the Ag-Cl bond description. The fitting parameters were the coordination numbers (CN), interatomic distances *R*, disorder factors σ^2 for metal-oxygen, metal-chloride and metal-metal bonds, as well as the correction to photoelectron reference energy ΔE_0. The amplitude reduction factor due to many-electron excitations (S_0^2 factor) was obtained in the fits of reference materials (corresponding metal foils). Due to the short length of the spectra acquired and, hence, limited resolution in R-space, we included only a single path to model the overlapping metal-metal contributions from the first two coordination shells in bcc-type iron. Fitting was carried out in R-space. The *R*-ranges used for fitting, and the k-ranges used for Fourier transformation, are reported in Table 3.

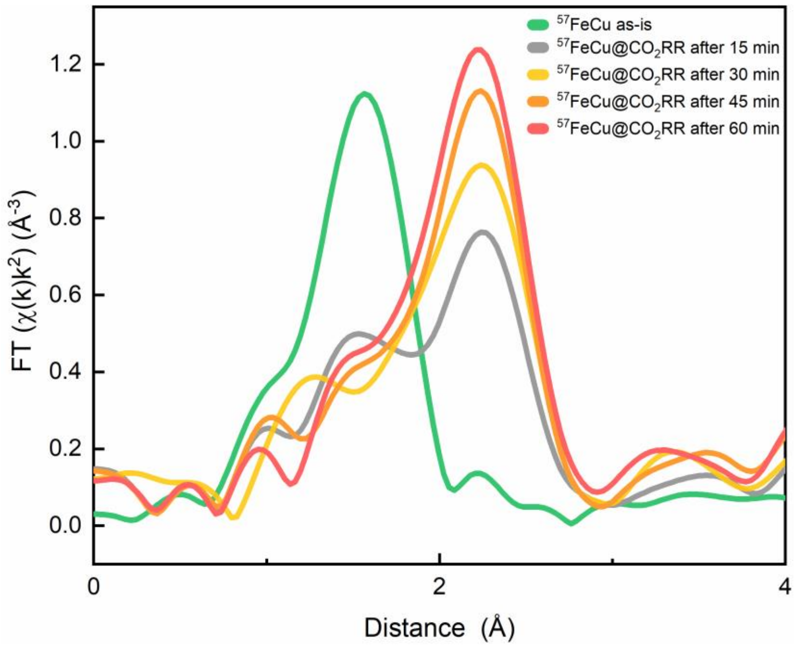

Figure S7: Fourier-transformed EXAFS data at different times after starting the measurement under CO_2RR conditions. The total measurement time was about 1 h. Individual spectra shown here correspond to a binned group of spectra over a time of 15 min each.

Table S3: Detailed fitting parameters: R-range, k-range and R_{bkg} (the frequency cutoff used to separate the background contribution) of the EXAFS fits.

Spectrum	R-range (Å)	k-range ($Å^{-1}$)	R_{bkg} (Å)
**CuK-edge in ^{57}FeCu as-is**	1.0-3.0	3.0-9.0	1.0
**Cu K-edge in ^{57}FeCu @ CO_2RR**	1.0-3.0	3.0-9.0	1.0
**Fe K-edge in ^{57}FeCu as-is**	1.0-3.0	3.0-9.0	1.0
**Fe K-edge in ^{57}FeCu @ CO_2RR**	1.0-3.0	3.0-9.0	1.0
**Ag K-edge in ^{57}FeAg as-is**	1.0-3.2	3.0-9.0	1.0
**Ag K-edge in ^{57}FeAg @ CO_2RR**	1.0-3.2	3.0-9.0	1.0
**Fe K-edge in ^{57}FeAg as-is**	1.0-3.0	3.0-9.0	1.0
**Fe K-edge in ^{57}FeAg @ CO_2RR**	1.0-3.0	3.0-9.0	1.0

Table S4: EXAFS Debye-Waller factor (σ^2) and fit quality ("R-factor", as reported by Artemis program) of the EXAFS fits.

	σ^2/Å^2	R-factor (%)
**^{57}FeAg as-is**		
**Ag-Cl**	0.018 ± 0.002	2.71
**Fe-O**	0.011 ± 0.003	1.96
**^{57}FeAg operando**		
**Ag-Ag**	0.009 ±- 0.001	2.86
**Fe-Fe**	0.008 ± 0.013	2.67
**Fe-O**	0.002 ± 0.005	2.04
**^{57}FeCu as-is**		
**Cu-O**	0.003 ± 0.002	1.99
**Fe-O**	0.023 ± 0.023	2.76
**^{57}FeCu operando**		
**Cu-Cu**	0.008 ± 0.015	2.53
**Cu-O**	0.006 ± 0.003	1.94
**Fe-Fe**	0.008 ± 0.004	2.03
**Fe-O**	0.004 ± 0.007	2.61

References

[1] J. R. Croy, S. Mostafa, J. Liu, Y.-h. Sohn, B. Roldan Cuenya, *Catal Lett* **2007**, *118*, 1-7.

[2] B. Roldan Cuenya, J. R. Croy, S. Mostafa, F. Behafarid, L. Li, Z. Zhang, J. C. Yang, Q. Wang, A. I. Frenkel, *J Am Chem Soc* **2010**, *132*, 8747-8756.

[3] G. Winter, D. W. Thompson, J. R. Loehe, in *Inorganic Syntheses*, **1973**, pp. 99-104.

[4] D. Nečas, P. Klapetek, *Open Phys* **2012**, *10*, 181-188.

[5] W. Sturhahn, *Hyperfine Interact* **2000**, *125*, 149-172.

[6] B. Ravel, M. Newville, *J Synchrotron Radiat* **2005**, *12*, 537-541.

[7] B. Roldan Cuenya, W. Keune, R. Peters, E. Schuster, B. Sahoo, U. v. Hörsten, W. Sturhahn, J. Zhao, T. S. Toellner, E. E. Alp, S. D. Bader, *Phys Rev B* **2008**, *77*, 165410.

[8] S. I. Zabinsky, J. J. Rehr, A. Ankudinov, R. C. Albers, M. J. Eller, *Phys Rev B* **1995**, *52*.
